# Management of malignant ureteric obstruction with ureteric stenting or percutaneous nephrostomy

**DOI:** 10.1093/bjs/znae035

**Published:** 2024-02-26

**Authors:** James Blackmur, Scottish Malignant Ureteric Obstruction Study Group, J P Blackmur, O Llewellyn, A Laird, S Teahan, R McLennan, S A McNeill, P Mariappan, L Kerr, J J Aning, N J Young, A Marzoug, D Hamilton, A Ng, D Fernando, L Drummond, P Holt, C Boyle, J S Shin, A Carrera, X W Lee, J Anderson, B Dreyer, J Krishnan, F Rodger, J Singh, C McCollum, C Boyle, C Zycinski, G M Nandwani, G Nabi, J Brush, J Taylor, J Keanie, P Granitsiotis, L Hayward, D McLaren, A Law, S G Kata, B G Thomas, F Al Jaafari

**Affiliations:** Department of Urology, University of Edinburgh, Institute of Genetics and Cancer, Western General Hospital, Edinburgh, UK

## Introduction

Malignant ureteric obstruction (MUO) is intrinsic or extrinsic obstruction of one or both ureters by malignancy. The incidence of MUO is poorly captured; however, has been reported in up to 4% of patients with advanced malignancy^1–3^. Unlike other oncological emergencies, such as malignant spinal cord compression, there is no standard approach to its management. Intervention to decompress the kidney(s) via percutaneous nephrostomy (PCN) or ureteric stenting (US) may be undertaken to facilitate treatment of urosepsis or significant electrolyte disturbance, for palliative treatment of intractable pain, or in an attempt to improve renal function and facilitate oncological treatment. These interventions, however, may be associated with morbidity, mortality, and have quality-of-life implications^4–14^.

Given the potential negative impact and unpredictable overall survival (OS) after intervention, it is important to determine which patients benefit most. Several small retrospective studies^15–19^ have demonstrated that survival in this cohort is poor. One study^15^ of 53 patients demonstrated that the primary cancer site, laterality, creatinine concentration at presentation, and whether the patient went on to receive oncological treatment predicted outcome after US for MUO (PLaCT score). Although this was subsequently validated in a 300-patient case series across 8 centres, critically this did not include patients managed with PCN, and the separation of prognosis into good, intermediate, and poor groups in that study is unlikely to provide the granularity of analysis needed to allow adequate patient counselling^20^.

The authors sought to define patient outcomes after US or PCN for MUO in a large multicentre national cohort, and to assess patient and clinical factors with respect to OS. A further aim was to develop and validate a scoring system using parameters available at the time of presentation to identify patients with short OS (90 days or less) in whom relief of obstruction may not be indicated.

## Methods

As an audit of standard of care, the study did not require specific ethical approval, in accordance with National Health Service Health Research Authority guidance. Caldicott Guardian approval was sought before local data collection. The study is reported in line with STROBE guidelines.

**Fig. 1:**
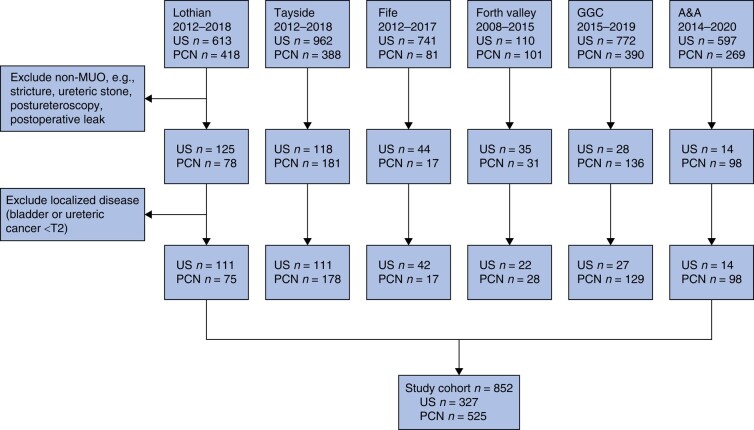
Study flow diagram Patients were identified by a search of procedure codes. GGC, greater glasgow and clyde; A&A, ayrshire and arran; US, ureteric stenting; PCN, percutaneous nephrostomy; MUO, malignant ureteric obstruction.

Consecutive patients with locally advanced or metastatic cancer undergoing primary US or PCN insertion for MUO from six health boards across Scotland between 2008 and 2020 were included in this study. Patients were identified locally as having undergone US or PCN insertion from prospectively recorded operation codes (*Supplementary material*). Relevant clinicopathological data were extracted to a standardized pro forma (*Supplementary material*). Data exclusions are shown in *Fig. 1*.

Primary outcomes were OS after decompression, and renal function at 3 months, specifically the proportion of the cohort with an estimated glomerular filtration rate (eGFR) over 45 ml per min per 1.73 m^2^, in accordance with the Kidney Disease Improving Global Outcomesk (KDIGO) class of initial renal failure^21^. This eGFR threshold was chosen as one that may allow chemotherapy to be considered. Secondary outcomes were return to oncological treatment (surgery, systemic therapy or radiotherapy, regardless of intent), and the development of a scoring system (Scottish MUO Score) to predict patients with a survival time of 90 days or less, and model its performance in prediction of overall survival at 30 days as well as 6 and 12 months. The performance of the Scottish MUO Score was compared with that of the PLaCT score^15,20^, which is used specifically in the setting of MUO, the modified Glasgow Prognostic Score (mGPS)^22,23^, and the C-reactive protein to albumin ratio (CRP : Alb)^24–31^.

Disease stage was broadly categorized as either locally advanced or metastatic, in accordance with the AJCC 8th edition for each cancer type. Locally advanced disease was defined as tumours of urological, gynaecological or gastrointestinal origin, radiologically staged as at least T2, causing intrinsic or extrinsic MUO owing to the mass effect of the primary tumour. The rationale for intervention was determined from contemporaneous medical records.

## Statistical analysis

All statistical analysis was performed using R version 4.2.0 (R Foundation for Statistical Computing, Vienna, Austria) and RStudio version 2022.02.3 (Posit, Boston, MA, USA). *P* < 0.050 was considered significant. Details of handling of missing variables, imputation, and model development are provided in the *Supplementary material*.

## Results

**Fig. 2:**
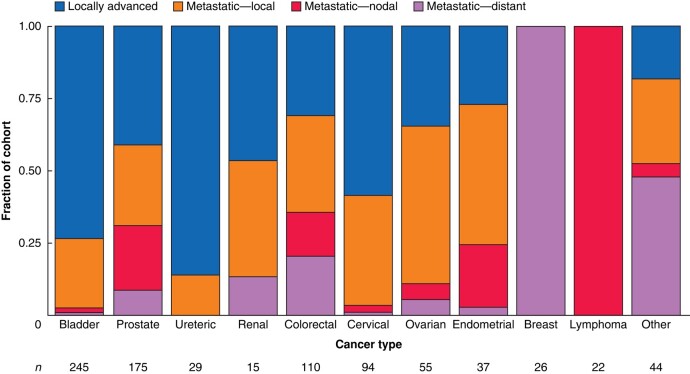
Nature of lesion causing obstruction for each cancer type, and whether the disease was locally advanced or metastatic at the time of intervention Metastatic disease is subdivided according to the nature of the lesion causing ureteric obstruction: local disease (including adjacent nodal stations contiguously involved with primary tumour), regional lymph nodes not in continuity with the primary tumour, or non-regional lymph nodes/distant metastases.

PCN or US was performed for MUO in 852 patients during the study interval, with a median follow-up of 7 (i.q.r. 2–20) months. Demographic and clinical data are shown in *Table 1*. The cancers were of urological origin in 466 patients (54.7%), gynaecological in 192 (22.5%), and gastrointestinal in 124 (14.6%). The nature of the disease causing ureteric obstruction is summarized in *Fig. 2*. Intervention was undertaken in patients 124 (14.6%) even though they had an eGFR exceeding 60 ml per min per 1.73 m^2^, with marked differences in the rates of initial US or PCN between centres (*Fig. S1*). Data were available regarding the primary rationale for intervention in 584 of the cohort (*Table 1*).

**Table 1:** Demographic and clinical data for patients included in the study

	No. of patients* (*n* = 852)
**Patient factors**	
Age at intervention (years), median (i.q.r.)	72 (62–79)
Sex	
Female	414 (48.6)
Male	438 (51.4)
Cancer type	
Bladder	245 (28.8)
Prostate	175 (20.5)
Ureteric	29 (3.4)
Renal	15 (1.8)
Colorectal	110 (12.9)
Cervical	94 (11.0)
Ovarian	55 (6.5)
Endometrial	37 (4.3)
Breast	26 (3.1)
Lymphoma	22 (2.6)
Other	44 (5.2)
Cancer status at time of intervention	
Locally advanced	410 (48.1)
Metastatic	442 (51.9)
**Radiological, haematological, and biochemical parameters**	
Laterality of hydronephrosis	
Unilateral	421 (49.4)
Bilateral	431 (50.6)
Haemoglobin (g/l), median (i.q.r.)	105 (93–118)
Missing	2 (0.2)
White cell count (× 10^9^/l), median (i.q.r.)	8.8 (6.7–11.8)
Missing	7 (0.8)
CRP (mg/l), median (i.q.r.)	57 (19–133)
Missing	62 (7.3)
Albumin (g/l), median (i.q.r.)	31 (25–36)
Missing	30 (3.5)
Corrected calcium (mmol/l), median (i.q.r.)	2.33 (2.24–2.43)
Missing	52 (6.1)
Sodium (mmol/l), median (i.q.r.)	137 (134–140)
Missing	2 (0.2)
Potassium (mmol/l), median (i.q.r.)	4.6 (4.1–5.1)
Missing	5 (0.6)
Creatinine (µmol/l), median (i.q.r.)	183 (116–346)
Missing	2 (0.2)
Renal function according to eGFR (ml per min per 1.73 m^2^)	
Normal (> 60)	124 (14.6)
3a (46–60)	115 (13.5)
3b (31–45)	161 (18.9)
4 (16–30)	201 (23.6)
5 (≤ 15)	249 (29.2)
Missing	2 (0.2)
**Regional health board**	
NHS Tayside	289 (33.9)
NHS Lothian	186 (21.8)
NHS GGC	156 (18.3)
NHS A&A	112 (13.1)
NHS Fife	59 (6.9)
NHS Forth Valley	50 (5.9)
**Indication for intervention**	
Acute kidney injury	337 (39.6)
Acute kidney injury + sepsis	30 (3.5)
Sepsis	25 (2.9)
Hydronephrosis on imaging/optimization for further intervention	182 (21.4)
Pain	10 (1.2)
Unknown†	268 (31.5)

*Values are *n* (%) unless otherwise indicated. †Data not available from National Health Service (NHS) greater glasgow and clyde (GGC) or NHS ayrshire and arran (A&A). CRP, C-reactive protein; eGFR, estimated glomerular filtration rate.

**Fig. 3:**
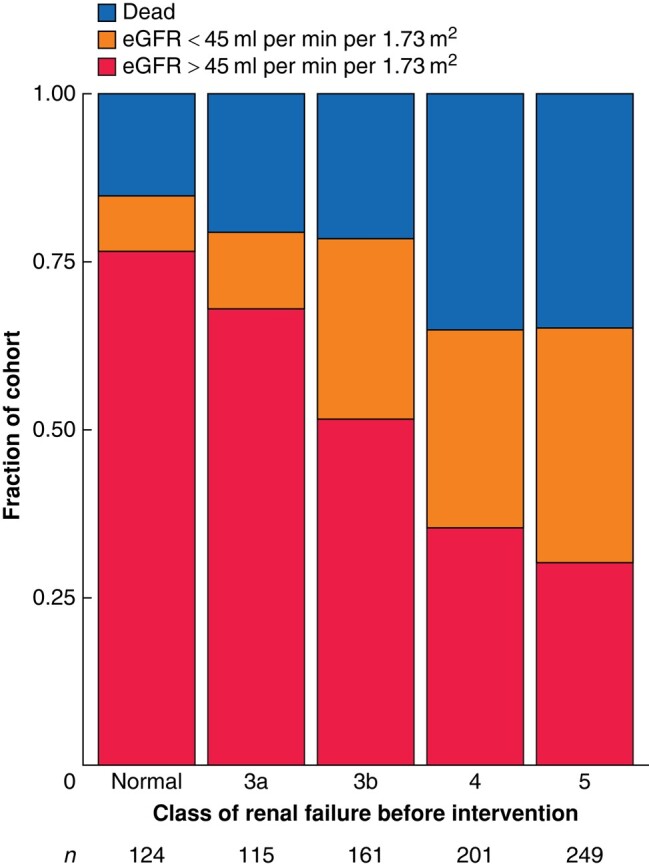
Relationship between outcome at 3 months and KDIGO class of renal failure before intervention Kidney Disease Improving Global Outcomes (KDIGO) class of renal failure was determined from the estimated glomerular filtration rate (eGFR). An eGFR of 45 ml per min per 1.73 m^2^ was chosen as the threshold that might allow future chemotherapy.

At 3 months after intervention, 378 patients (44.4%) had over 20% improvement in creatinine level, whereas 227 (26.6%) had no improvement. Renal function at 3 months stratified by class of initial renal failure is shown in *Fig. 3*. The proportion of individuals with an eGFR of at least 45 ml per min per 1.73 m^2^ at 3 months varied significantly according to initial class of renal failure (*P* < 0.001), with a significant proportion of patients with an eGFR in the range of chronic kidney disease (CKD) 5, CKD4, and CKD3b showing an improvement in eGFR to 45 ml per min per 1.73 m^2^ or higher (30.1, 35.3, and 51.6% respectively). Of patients with an eGFR in the normal or CKD3a range before intervention, 9.6% had a reduction in eGFR to below 45 ml per min per 1.73 m^2^.

During the study interval, 758 patients (89.0%) died with a median survival of 6 (i.q.r. 2–14) months. A total of 72 individuals (8.5%) died within 30 days of intervention, 236 (27.7%) within 90 days, 373 (43.8%) within 6 months, and 528 (62.0%) within 1 year. Oncological treatment (surgery, systemic therapy or radiotherapy) was administered after decompression to 366 patients (43.0%). Of the patients who did not proceed to oncological treatment, 72 (14.8%) underwent decompression because of hydronephrosis on imaging and/or for optimization for further intervention, 244 (50.2%) underwent decompression owing to acute kidney injury (AKI) and/or sepsis, 2 (0.4%) because of pain, and in 168 (34.6%) the indication unknown.

Patient, radiological, haematological, and biochemical parameters relevant to OS were assessed by Cox regression (*Table 2*). In the adjusted model, age, metastatic disease at time of intervention, bilateral hydronephrosis, low haemoglobin, high potassium, high white cell count, and high CRP level were associated with worse OS. Although there were differences in OS based on cancer type on unadjusted analysis, after adjustment this only held true for prostate cancer and lymphoma.

**Table 2:** Factors associated with overall survival assessed by unadjusted and adjusted multivariable Cox regression analyses

	Unadjusted analysis		Adjusted analysis	
	HR	*P*	HR	*P*
Age	**1.01 (1.01, 1.02)**	**< 0.001**	**1.01 (1.00, 1.02)**	**0.004**
**Sex**				
Female	1.00 (reference)		1.00 (reference)	
Male	1.10 (0.95, 1.27)	0.19	0.98 (0.79, 1.21)	0.84
**Cancer type**				
Bladder	1.00 (reference)		1.00 (reference)	
Prostate	0.90 (0.74, 1.11)	0.32	**0.73 (0.56, 0.93)**	**0.01**
Ureteric	**0.66 (0.43, 0.99)**	**0.046**	1.07 (0.68, 1.67)	0.77
Renal	**0.55 (0.31, 0.99)**	**0.045**	1.08 (0.53, 2.23)	0.83
Colorectal	1.00 (0.79, 1.26)	0.97	0.88 (0.66, 1.17)	0.38
Cervical	**0.56 (0.43, 0.73)**	**< 0.001**	0.73 (0.51, 1.03)	0.07
Ovarian	0.95 (0.70, 1.29)	0.76	0.89 (0.61, 1.30)	0.54
Endometrial	0.81 (0.56, 1.17)	0.27	0.69 (0.44, 1.06)	0.09
Breast	1.08 (0.72, 1.62)	0.72	0.86 (0.54, 1.38)	0.53
Lymphoma	**0.48 (0.28, 0.81)**	**0.006**	**0.45 (0.24, 0.85)**	**0.02**
Other	1.15 (0.82, 1.61)	0.43	0.93 (0.61, 1.41)	0.73
**Cancer status at time of intervention**				
Locally advanced	1.00 (reference)		1.00 (reference)	
Metastatic	**1.43 (1.23, 1.65)**	**< 0.001**	**1.50 (1.24, 1.82)**	**< 0.001**
**Hydronephrosis**				
Unilateral	1.00 (reference)		1.00 (reference)	
Bilateral	**1.56 (1.35, 1.80)**	**< 0.001**	**1.30 (1.09, 1.56)**	**0.004**
Haemoglobin (g/l)	**0.99 (0.98, 0.99)**	**< 0.001**	**0.99 (0.99, 1.00)**	**0.04**
White cell count (x10^9^/l)	**1.04 (1.03, 1.06)**	**< 0.001**	**1.03 (1.01, 1.04)**	**0.001**
CRP (mg/l)	**1.00 (1.00, 1.00)**	**< 0.001**	**1.00 (1.00, 1.00)**	**0.02**
**Renal function**				
Normal	1.00 (reference)		1.00 (reference)	
3a	1.20 (0.91, 1.60)	0.20	0.91 (0.65, 1.27)	0.58
3b	**1.67 (1.29, 2.17)**	**< 0.001**	1.33 (0.98, 1.81)	0.07
4	**1.72 (1.34, 2.21)**	**< 0.001**	1.21 (0.90, 1.62)	0.21
5	**2.03 (1.60, 2.58)**	**< 0.001**	1.11 (0.81, 1.52)	0.51
Sodium (mmol/l)	**0.96 (0.95, 0.98)**	**< 0.001**	0.99 (0.97, 1.01)	0.19
Potassium (mmol/l)	**1.39 (1.25, 1.55)**	**< 0.001**	**1.25 (1.10, 1.41)**	**< 0.001**
Albumin (g/l)	**0.99 (0.98, 1.00)**	**0.005**	1.00 (0.98, 1.01)	0.41
Corrected calcium (mmol/l)	1.13 (0.71, 1.80)	0.62	1.23 (0.75, 2.02)	0.42
**Region**				
NHS Tayside	1.00 (reference)		1.00 (reference)	
NHS Lothian	1.14 (0.94, 1.38)	0.20	1.00 (0.80, 1.26)	0.98
NHS GGC	**0.77 (0.62, 0.95)**	**0.02**	**0.65 (0.51, 0.82)**	**< 0.001**
NHS A&A	1.05 (0.84, 1.32)	0.68	0.89 (0.68, 1.15)	0.37
NHS Fife	**0.71 (0.53, 0.96)**	**0.02**	0.71 (0.49, 1.03)	0.08
NHS Forth Valley	**0.47 (0.34, 0.66)**	**< 0.001**	**0.48 (0.32, 0.74)**	**0.001**

Values in parentheses are 95% confidence intervals. HRs for continuous variable are shown per unit increase. 127 patients with missing variables were excluded. CRP, C-reactive protein; GGC, greater glasgow and clyde; A&A, ayrshire and arran.

### Development of the Scottish MUO Score

Demonstration that cancer type is not the primary driver of survival provides evidence supporting the use of pan-cancer scores in patients with MUO. The presence and distribution of missing variables were assessed, and were then imputed, as described in the *Supplementary material* and shown in *Fig. S2*. After development, comparison of clinical and biochemical parameters between discovery and validation cohorts is reported in *Table S1*.

Stepwise regression identified that a model including age, CRP, haemoglobin, creatinine, potassium, sodium, cancer type, metastatic disease, and bilateral hydronephrosis was associated with the lowest information loss in determining OS in the discovery cohort. K-fold cross-validation was then used to determine the weightings of each of these variables in a predictive model for 90-day survival (*Supplementary material*). This model is simplified for general use at: https://webapps.igc.ed.ac.uk/world/research/muo_calculator/. As well as calculating the Scottish MUO Score, the website also provides prediction of 30-day, 90-day, 6-month, and 12-month OS based on results from the discovery cohort (*Supplementary material*).

### Model performance

**Fig. 4:**
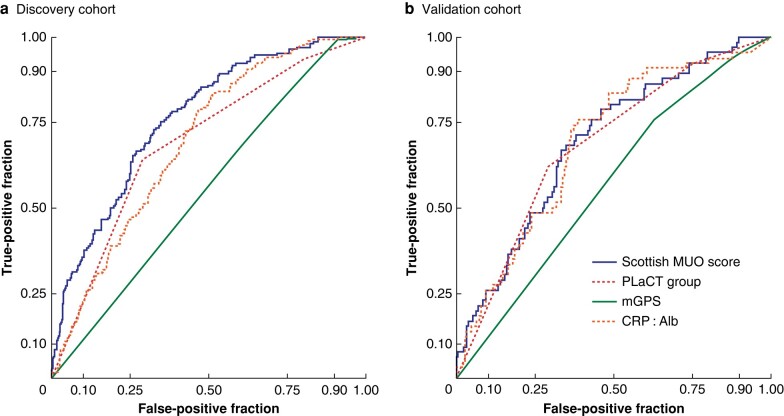
Receiver operating characteristic (ROC) curves for 3-month survival (90 days or less) according to scoring system **a** Discovery and **b** validation cohort. MUO, Malignant ureteric obstruction; mGPS, modified Glasgow Prognostic Score; CRP : Alb, C-reactive protein to albumin ratio.

Model performance was assessed in discovery and validation cohorts by means of receiver operating characteristic (ROC) curves for the primary outcome of 90-day OS (*Fig. 4* and *Table 3*), along with secondary outcomes of 6-month (*Table S2a*), 1-year (*Table S3a*), and 30-day OS (*Table S4a*).

**Table 3:** Comparison of model performance for 3-month survival (90 days or less) in discovery and validation cohorts

Prognostic scoring system	AUC	*P* (pairwise comparison with Scottish MUO Score)	Accuracy (NPV)	Precision (PPV)	Recall (Sensitivity)	Specificity
**Discovery cohort**						
Scottish MUO Score	0.78 (0.02)	–	0.75	0.76	0.18	0.98
PLaCT	0.69 (0.02)	0.001	0.89	0.32	0.94	0.20
mGPS	0.55 (0.02)	< 0.001	0.98	0.30	0.99	0.10
CRP : Alb	0.69 (0.02)	< 0.001	0.93	0.34	0.95	0.27
**Validation cohort**						
Scottish MUO Score	0.71 (0.04)	–	0.77	0.45	0.20	0.92
PLaCT	0.69 (0.03)	0.58	0.90	0.30	0.92	0.25
mGPS	0.57 (0.03)	0.002	0.86	0.27	0.95	0.10
CRP : Alb	0.69 (0.03)	0.47	0.90	0.31	0.91	0.30

Values in parentheses are standard errors. AUC, area under the curve; MUO, malignant ureteric obstruction; NPV, negative predictive value; PPV, positive predictive value; mGPS, modified Glasgow Prognostic Score; CRP : Alb, C-reactive protein to albumin ratio.

To explore differences in specificity and sensitivity between scores, OS was compared in the discovery cohort for each of the scoring systems (*Tables 4*, *S2b*, *3b*, and *4b*). A range of OS outcomes was represented across deciles of the Scottish MUO Score (1.7–73.3% died within 90 days, and 15.0–85.0% died within 6 months), whereas CRP : Alb, PLaCT score, and mGPS were less able to personalize OS assessment as they had a narrower range of outcome predictions.

**Table 4:** Comparison of 90-day overall survival (90 days or less) in discovery cohort for each scoring system

Decile	Scottish MUO Score		CRP : Alb		Group	PLaCT Score		mGPS	
	*n*	Died within 90 days	*n*	Died within 90 days		n*	Died within 90 days	n*	Died within 90 days
1	60	1 (1.7)	60	1 (1.7)	Good	96 (16.1)	11 (11.5)	40 (6.7)	1 (2.5)
2	59	4 (6.8)	59	7 (11.9)					
3	60	9 (15.0)	60	10 (16.7)					
4	59	5 (8.5)	59	11 (18.6)	Intermediate	268 (45.0)	50 (18.7)	150 (25.2)	43 (28.9)
5	60	16 (26.7)	60	23 (38.3)					
6	59	18 (30.5)	59	21 (35.6)					
7	59	21 (35.6)	59	21 (35.6)	Poor	231 (38.8)	109 (47.2)	405 (68.1)	126 (31.1)
8	60	28 (46.7)	60	24 (40.0)					
9	59	24 (40.7)	59	24 (40.7)					
10	60	44 (73.3)	60	28 (46.7)					

Values are *n* (%); *percentage of cohort. Scottish malignant ureteric obstruction (MUO) Score and C-reactive protein to albumin ratio (CRP : Alb) are continuous scales, and are grouped by decile. PLaCT score and modified Glasgow Prognostic Score (mGPS) utilize three prognostic groups.

## Discussion

This study analysed the largest detailed cohort of patients undergoing intervention for MUO, demonstrating across a multicentre national cohort that there is variability in the use of PCN and US. These differences highlight the uncertainty clinicians face regarding if, when, and how to intervene in MUO. It is clear, however, that MUO is associated with poor prognosis, with 89% of patients dying during follow-up and the majority having no improvement in renal function or receiving further oncological treatment.

Although prognosis is poor overall (including 8.5% of patients surviving less than 30 days), there is also a large group (38%) surviving for over 1 year after intervention. To differentiate these groups, clinical factors associated with worse OS were identified. The presence of metastatic disease had a negative impact on survival. Although it was not possible to assess such factors in the present study, disease stage and nature of obstruction are likely to affect treatment options, which will be the focus of future work. Nonetheless, the presence or absence of metastatic disease is a key stratifier in the MUO Score. In adjusted Cox regression analysis, only prostate cancer and lymphoma were associated with OS independently of the presence or absence of metastatic disease. This suggests that MUO may be a marker of advanced disease independent of the underlying tumour biology of different cancer types, supporting the use of a pan-cancer survival score in patient counselling before intervention.

The Scottish MUO Score developed and validated in this study includes parameters readily accessible in routine clinical practice, and the online platform allows straightforward calculation. It is also noted that, to a clinician and patient, life expectancy may be of greater relevance. Uniquely, the online platform uses routine clinical parameters to provide an assessment of 30-day, 90-day, 6-month, and 12-month OS from the discovery cohort, providing a personalized assessment of survival to guide patient counselling and decision-making.

The Scottish MUO Score compares favourably with other prognostic scores^15,22,23^. The attractiveness of the other scores is their simplicity, but they may fail to adequately discriminate between differences in outcomes^15,20^. To a patient contemplating intervention, the ability to determine whether they are going to be alive at defined time points is of paramount importance, and the Scottish MUO Score allows this. The PLaCT score and mGPS classified a high proportion of individuals in the poor prognostic groups. Although this leads to high sensitivity, patients may outlive this predicted life expectancy and might have been counselled against intervention. The lack of resolution about an individual’s prognosis makes these scores unhelpful in a real-world clinical setting. The Scottish MUO Score discriminated patients with better OS; many patients with a low CRP : Alb died within 90 days (19.2% discovery cohort and 12.7% validation cohort CRP : Alb below 2), but this occurred only in 2 of 91 patients (2.2%) in the combined cohorts with a Scottish MUO Score of less than −3.0.

Decision-making in this area is complex, and a rationale for intervention for palliation remains in certain patients even when survival is expected to be poor. Potential immediately life-threatening indications, including sepsis and AKI, were present in 9.4 and 57.6% of the patients respectively, where intervention may be justified in the face of imminent death, even with the prospect of poor oncological outcomes. Indeed, although most patients did not have a significant improvement in renal function, there was a sizeable proportion of patients in this cohort with an arguably life-threatening eGFR of less than 15 ml per min per 1.73 m^2^ who showed an improvement to over 45 ml per min per 1.73 m^2^. However, an understanding of the survival benefit with intervention is important. Understanding potential benefits allows patient-centred discussion of the balance of risks and benefits, particularly in view of the morbidity associated with intervention^3–13^. Even among teams unfamiliar with the individual patient, the Scottish MUO Score can aid this discussion. Although the proportion of patients in this cohort who received further oncological treatment is similar to that in previous reports^32^, the data do not allow assessment of quality-of-life or outcomes without intervention, which is the subject of an ongoing prospective study.

Although the inherent bias of case (and procedure) selection between health boards is likely to at account least partly for the reported differences in survival, the heterogeneous approach to management captured in this multicentre study is a particular strength. As a retrospective review, however, it was not possible to ascertain whether oncological treatment was completed, or to distinguish whether treatment was undertaken with curative or palliative intent. Missing from this cohort (and in other studies to date) are the patients who do not go on to receive intervention, and the rationale for this decision. The present study is much larger than previous studies of patients with MUO, but it remains underpowered to subclassify each cancer type by stage or grade to fully appreciate their effect on OS. Further work to look at cancer-specific outcomes is warranted, particularly to determine whether bespoke scores for each cancer type could give additional prognostic information beyond the proposed pan-cancer Scottish MUO Score. Furthermore, differences in stage classification between cancers and their associated treatment options prevented their inclusion in the model, although this information could potentially be included in larger individual-cancer cohorts. The score also requires adequately powered external validation, particularly given that imputation was used for missing variables. Although definitions of curative or palliative treatment vary between specialties and across cancer types, further subgroup analyses would be of interest, and are the subject of an ongoing prospective study that also examines ethnicity, co-morbidity, readmission rates, rationale for the modality of decompression chosen, and quality of life.

MUO is a marker of advanced disease and survival in these patients is poor. Differences in approach to management and outcomes across Scottish institutions highlight the uncertainty in optimal management of this condition. Although there will always be a specific rationale for intervention in patients with MUO, improvement in renal function or progression to additional oncological treatments is not guaranteed. Understanding survival after intervention for MUO is key to decision-making regarding whether to perform US or PCN, and the Scottish MUO Score (available at https://webapps.igc.ed.ac.uk/world/research/muo_calculator/) can predict survival outcomes, aid patient counselling, and help deliver a patient-centred approach to treatment.

## Collaborators

Scottish Malignant Ureteric Obstruction Study Group: J.P. Blackmur, O. Llewellyn, A. Laird, S. Teahan, R. McLennan, S.A. McNeill, P. Mariappan, L. Kerr, J.J. Aning, N.J. Young, A. Marzoug, D. Hamilton, A. Ng, D. Fernando, L. Drummond, P. Holt, C. Boyle, J.S. Shin, A. Carrera, X.W. Lee, J. Anderson, B. Dreyer, J. Krishnan, F. Rodger, J. Singh, C. McCollum, C. Boyle, C. Zycinski, G.M. Nandwani, G. Nabi, J. Brush, J. Taylor, J. Keanie, P. Granitsiotis, L. Hayward, D. McLaren, A. Law, S.G. Kata, B.G. Thomas, F. Al Jaafari.

## Supplementary Material

znae035_Supplementary_Data

## Data Availability

Anonymized data and analysis code are available on request and Caldicott Guardian approval.
