# Supplementary material for: Management of malignant ureteric obstruction with ureteric stenting or percutaneous nephrostomy
Source: Br J Surg. 2024 Feb 26;111(2):znae035. doi: 10.1093/bjs/znae035 (PMC10895405; doi:10.1093/bjs/znae035)
Supplement: znae035_Supplementary_Data [file znae035_supplementary_data.docx]

**A national experience managing malignant ureteric obstruction with ureteric stent or nephrostomy in Scotland**

Scottish Malignant Ureteric Obstruction Study Group

**Corresponding author.** Mr James Blackmur, Clinical Lecturer in Urology and Honorary Specialty Trainee, Department of Urology, University of Edinburgh, Western General Hospital, Crewe Road South, Edinburgh, EH4 2XU. [james.blackmur1@nhs.net](mailto:james.blackmur1@nhs.net)

ORCID 0000-0001-7267-837X

Twitter: @BlackmurJames

**Supplementary Materials - Index**

| **Supplementary Methods** |  |
| --- | --- |
| Patient identification | *pg. 2* |
| Missing variables  Model development | *pg. 2*  *pg. 2* |
| **Supplementary Results** |  |
| Scottish MUO Score variable weighting | *pg. 4* |
| **Supplementary Figures and Tables** |  |
| *Supplementary Figure 1* | *pg. 5* |
| *Supplementary Figure 2* | *pg. 6* |
| *Supplementary Table 1* | *pg. 7* |
| *Supplementary Table 2* | *pg. 8* |
| *Supplementary Table 3* | *pg. 9* |
| *Supplementary Table 4* | *pg. 10* |
| **References** | *pg. 11* |
|  |  |

**Supplementary Methods**

*Patient identification*

Patients were identified as having undergone stent or nephrostomy insertion by search of prospectively recorded operation codes (OPCS Classification of Interventions and Procedures version 4) related to stent or nephrostomy in the Opera Theatre Management System (Centricity), Operating Room Scheduling Office System (ORSOS, Per-Se Technologies, Inc.) or Radiology Information System (RIS). Electronic health records were screened locally to confirm the patient had undergone US or PCN insertion for MUO. Data were extracted regarding relevant clinico-pathological parameters: age at intervention, gender, cancer type, uni- or bi-lateral hydronephrosis noted on ultrasound or computed tomography (CT) in radiology report, whether the disease was locally advanced or metastatic at the time of intervention (and in the case of metastatic disease, whether the lesion causing obstruction represents local disease [including adjacent nodal stations contiguously involved with primary tumour], regional lymph nodes per cancer type not in continuity with the primary tumour, or non-regional lymph nodes/distant metastases), haemoglobin (Hb), white cell count (WCC), C-reactive protein (CRP), creatinine (Cr), estimated glomerular filtration rate (eGFR, calculated by clinical biochemistry departments locally using MDRD equation), sodium (Na), potassium (K), albumin (Alb), calcium (Corr ca, corrected for albumin). Haematological, biochemical and radiological findings were the most recent in the month prior to intervention. Technical considerations of retrograde US, antegrade US, PCN and their impact on overall survival or stent failure, and conversion between US to PCN or vice versa were not considered in this analysis. Patients were anonymised, and data then exported by Microsoft Excel files for analysis in NHS Lothian/University of Edinburgh in R(v4.2.0) and RStudio(v2022.02.3). Out-of-hours Interventional radiology (i.e. PCN) services are provided to NHS Ayrshire and Arran (A&A) and NHS Forth Valley by NHS Greater Glasgow and Clyde (GGC), and to NHS Fife by NHS Tayside. Patients were classified according to the Health Board where intervention was undertaken. After assessing for significant collinearity (Pearson r>0.6 and FDR p<0.05), Cox-regression analysis was performed with the *survival* (v3.3.1)(1), *survminer* (v0.4.9)(2) and *finalfit* (v1.0.4)(3) packages.

*Missing variables*

The presence of missing variables was assessed using the *finalfit*(3) package. Imputation was undertaken using the k-Nearest Neighbour (kNN) method of the *VIM* package (v6.1.1)(4) (using k=5; distance variables included age, gender, haemoglobin, white cell count, C-reactive peptide, eGFR, sodium, potassium, albumin and corrected calcium). Complete data were present regarding survival outcome, age, cancer type, laterality of hydronephrosis, and Hb. Five patients had missing WCC, 60 missing CRP, three missing K, 28 missing Alb and 50 missing Corr Ca. One hundred and five individuals had a single missing variable. Nineteen individuals were missing two variables (11 missing CRP and Corr Ca, two missing CRP and Albumin, three missing WCC and K, one missing WCC and CRP, one missing WCC and Corr Ca and one missing Corr Ca and Alb), and one missing three variables (CRP, Alb, Corr Ca).

*Model development*

Normal distribution of age and haematological/biochemical parameters were demonstrated not to be normally distributed by histogram and Shapiro-Wilk test. These parameters were log_e_ transformed prior to further analysis to reduce the impact of outlying values. With a view to relatively straightforward future use of a clinical predictive tool, log_e_ transformation [log_e_(parameter+1)] was chosen in preference to either min-max or Z-score standardisation.

In order to develop and then validate the Scottish MUO Score, the whole dataset was randomly split into discovery (70%) and validation (30%) cohorts. This method was chosen in preference to allocating Health Boards to discovery and validation cohorts given potential differences in approach between centres. Stepwise variable selection by **Akaike Information Criterion** (AIC, forward and backward) was used to identify the optimal Cox regression model associated with overall survival in the discovery cohort using the *MASS* package(v 7.3.58.1(5)). Factors included in that base model were chosen as objective measures that would be available to the clinician at the time of considering possible intervention: age, gender, cancer type, presence of metastatic disease, presence of uni- or bi-lateral hydronephrosis, along with haematological and biochemical parameters. Assessment of performance status, whether the subsequent intervention was uni- or bi-lateral, initial stent or nephrostomy, and whether the patient went on to receive further treatment were not included in the model. Ethnicity data were not collected. Assessment of performance status is complex and can be acutely altered, but is a strong predictor of future management and survival however was unable to assess retrospectively

Weightings of variables in the model were determined using k-fold cross validation by the *caret* package (v6.0.92)(6). Given the relatively large sample size and the desire for model selection, k-fold cross validation (k=10) was used in preference to bootstrapping. A generalised linear model was utilised and the optimal model selected on the basis of ROC. AUC, specificity and sensitivity for each of the 10-folds are quoted below. Scottish MUO Score was then calculated for each individual in both discovery and validation cohorts. PLaCT(7, 8), mGPS(9, 10) and CRP:Alb were calculated in both discovery and validation cohorts. Model performance in discovery and validation cohorts were compared by ROC curves using the *plotROC(v2.3.0)(11)* and *pROC(v1.18.0)(12)* packages, and by comparison of Area Under the curve (AUC), sensitivity, specificity, negative predictive value (NPV) and positive predictive value (PPV).

An additional prognostic score in MUO has been proposed by Cordeiro et al(13) by study of a relatively small cohort from two institutions in Brazil and not adequately externally validated. In that study ≥4 events related to malignancy and ECOG PS ≥2 were associated with worse overall survival, and the presence of these factors were used to split their cohort into good, intermediate or poor prognostic groups. In addition to the issues of lack of granularity, determining performance status in patients who have had an acute deterioration may be problematic and the definition of “events related to malignancy” is unclear and open to wide interpretation, particularly across different cancer types. Unfortunately this information was not available for our cohort and therefore direct comparison with this score was not possible.

Scottish MUO score in the discovery cohort was split into deciles, and the percentage of patients who died within 30, 90, 182 and 365 days in each decile were determined. Linear regression was used to assess the association of median Scottish MUO score per decile with percentage change of survival at those time points.

**Supplementary Results**

**Scottish MUO Score variable weighting**

Scottish MUO Score= 23.897022 + ((log_e_(Age+1))* 1.566178) + ((log_e_(CRP+1))* 0.583499)

+ ((log_e_(Hb+1))*- 1.147219) + (log_e_(Cr+1))* -0.187129) + ((log_e_(K+1))* 1.932691)

+ ((log_e_(Na+1))*- 6.392649)

[+0 if bladder cancer, -0.499384 prostate cancer, -0.330058 ureteric cancer,

+0.296135 renal cancer, -0.118341 colorectal cancer, -1.294641 cervical cancer, +0.007457 endometrial cancer, +0.123786 ovarian cancer,

+0.147122 breast cancer, -1.227878 lymphoma or +0.927012 if other origin]

[+0.524943 if bilateral hydronephrosis present]

[+0 if locally advanced , +0.879136 if metastatic at time of diagnosis MUO]

**This model derived the following ROC metrics across the 10-folds in the discovery cohort:**

| **Fold** | **AUC** | **Sensitivity** | **Specificity** |
| --- | --- | --- | --- |
| 1 | 0.76 | 0.90 | 0.35 |
| 2 | 0.74 | 0.81 | 0.41 |
| 3 | 0.74 | 0.93 | 0.24 |
| 4 | 0.71 | 0.91 | 0.35 |
| 5 | 0.81 | 0.93 | 0.29 |
| 6 | 0.77 | 0.88 | 0.41 |
| 7 | 0.81 | 0.93 | 0.24 |
| 8 | 0.79 | 0.95 | 0.35 |
| 9 | 0.72 | 0.84 | 0.18 |
| 10 | 0.60 | 0.86 | 0.18 |

**Equations derived in the Discovery cohort to assess the percentage of patients who were alive with a particular Scottish MUO Score.**

Percentage alive at 90 days= 52.511 + (Scottish MUO score*-15.723). R^2^ of model 0.84.

Percentage alive at 182 days= 34.360 + (Scottish MUO score*-16.767). R^2^ of model 0.93.

Percentage alive at 1 year= 19.820 + (Scottish MUO score*-15.454). R^2^ of model 0.94.

Percentage alive at 30 days=83.800 + (Scottish MUO score*-5.581). R^2^ of model 0.69.

**Supplementary Figures and Tables**


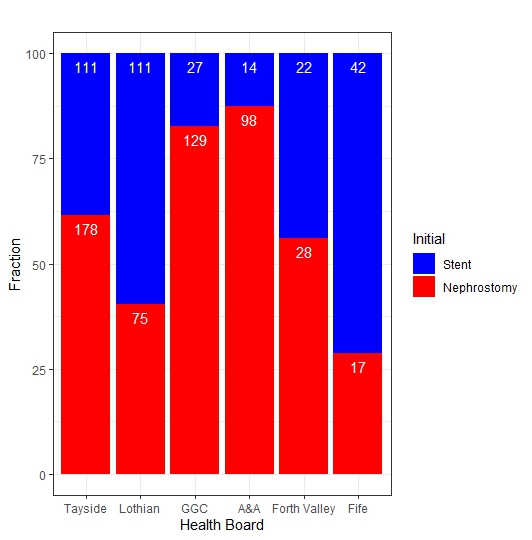


*Supplementary Figure 1: Differences in approach to initial management between centres (Pearson Chi-squared p-value <2.2e-16) labelled by initial treatment modality for each Health Board.*


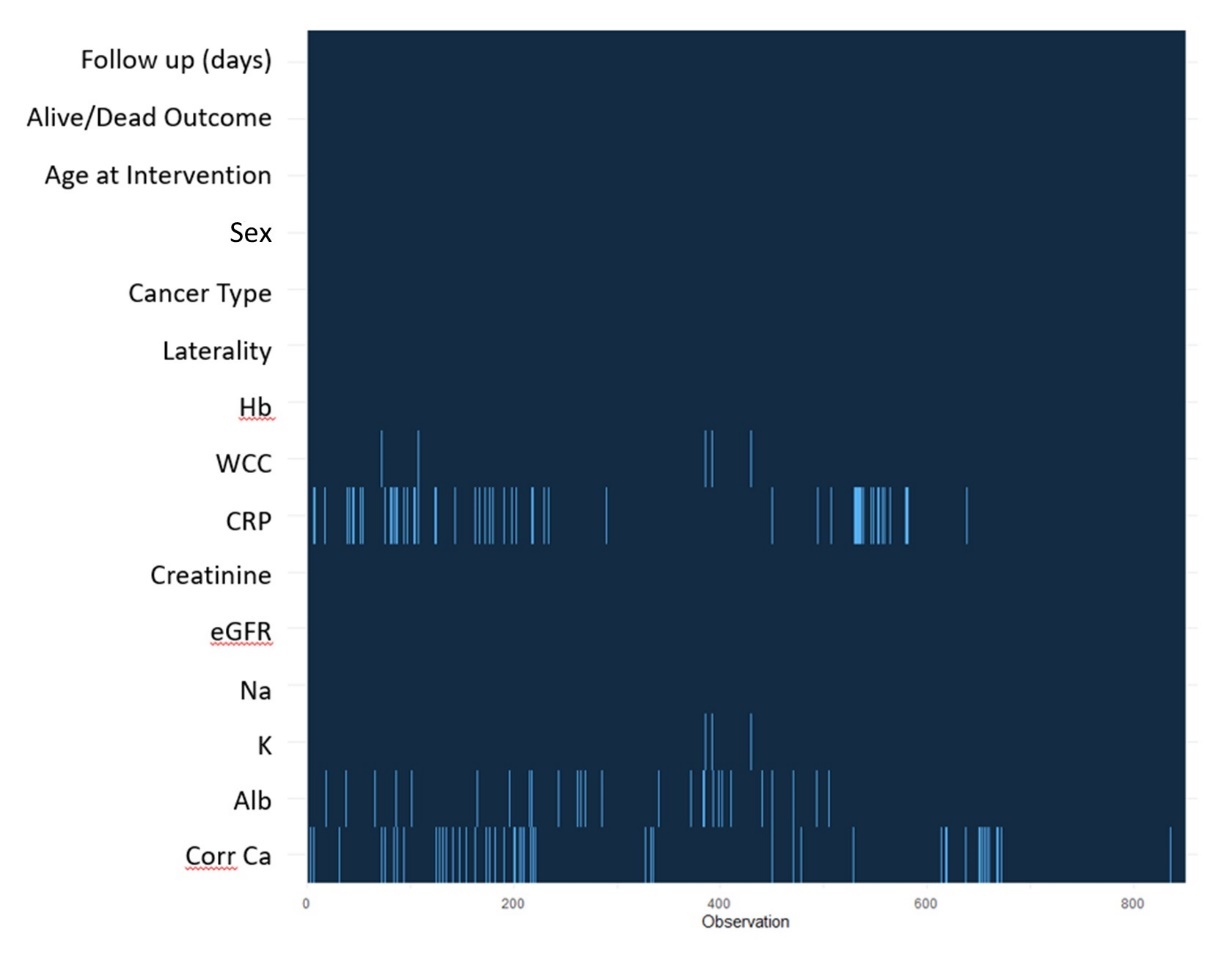


*Supplementary Figure 2: Distribution of missing data in 850 cases prior to imputation (missing data marked in light blue). (Hb Haemoglobin, WCC White cell count, CRP C-reactive protein, eGFR estimated glomerular filtration rate, Na Sodium, K Potassium, Alb Albumin, Corr Ca Corrected calcium).*

| *Supplementary Table 1: Comparison of demographic and clinical variables in the discovery and validation cohorts. Continuous variables were assessed by Mann-Whitney U test, while discrete variables by Chi-squared test.*   \| Variable \| Descriptors \| \| Discovery \| Validation \| p-value \| \| --- \| --- \| --- \| --- \| --- \| --- \| \| Age \| Median (IQR) \|  \| 72 (63-79) \| 71(62-80) \| 0.58 \| \| Sex \| N (%) \| Female \| 285 (47.9) \| 127 (49.8) \| 0.66 \| \| Male \| 310 (52.1) \| 128 (50.2) \| \| Cancer Type \| N (%) \| Bladder \| 186 (31.3) \| 59 (23.1) \| 0.20 \| \| Prostate \| 127 (21.3) \| 49 (19.2) \| \| Ureteric \| 14 (2.4) \| 15 (5.9) \| \| Renal \| 10 (1.7) \| 5 (2.0) \| \| Colorectal \| 75 (12.6) \| 34 (13.3) \| \| Cervical \| 60 (10.1) \| 32 (12.5) \| \| Ovarian \| 37 (6.2) \| 17 (7.1) \| \| Endometrial \| 24 (4.0) \| 13 (5.1) \| \| Breast \| 17 (2.9) \| 9 (3.5) \| \| Lymphoma \| 14 (2.4) \| 8 (3.1) \| \| Other \| 31 (5.2) \| 13 (5.1) \| \| Cancer locally advanced or metastatic (nodal or distant) at time of intervention \| N (%) \| Locally advanced \| 297 (49.9) \| 113 (44.3) \| 0.15 \| \| N (%) \| Metastatic \| 298 (50.1) \| 142 (55.7) \| \| Hydronephrosis \| N (%) \| Unilateral \| 283 (47.6) \| 138 (54.1) \| 0.09 \| \| Bilateral \| 312 (52.4) \| 117 (45.9) \| \| Hb \| Median (IQR) \|  \| 104 (93-118) \| 106 (95-120) \| 0.13 \| \| WCC \| Median (IQR) \|  \| **9.0 (6.8-12.1)** \| **8.4 (6.5-11.2)** \| **0.05** \| \| CRP \| Median (IQR) \|  \| 53 (20-127) \| 57 (17-136) \| 0.79 \| \| Renal function \| N (%) \| Normal \| 86 (14.4) \| 38 (14.9) \| 0.92 \| \| 3° \| 79 (13.3) \| 36 (14.1) \| \| 3b \| 111 (18.7) \| 50 (19.6) \| \| 4 \| 146 (24.5) \| 55 (21.6) \| \| 5 \| 173 (29.1) \| 76 (29.8) \| \| Na \| Median (IQR) \|  \| 137 (134-140) \| 138 (135-140) \| 0.16 \| \| K \| Median (IQR) \|  \| 4.5 (4.1-5.1) \| 4.6 (4.2-5.0) \| 0.35 \| \| Alb \| Median (IQR) \|  \| 31 (25-36) \| 32 (26-36) \| 0.37 \| \| Corrected ca \| Median (IQR) \|  \| 2.33 (2.24-2.42) \| 2.33 (2.25-2.42) \| 0.81 \| \| Region \| N (%) \| NHS Tayside \| 200 (33.6) \| 89 (34.9) \| 0.99 \| \| NHS Lothian \| 130 (21.8) \| 56 (22.0) \| \| NHS GGC \| 107 (18.0) \| 47 (18.4) \| \| NHS A&A \| 79 (13.3) \| 33 (12.9) \| \| NHS Forth Valley \| 37 (6.2) \| 13 (5.1) \| \| NHS Fife \| 42 (7.1) \| 17 (6.7) \| |
| --- | --- | --- | --- | --- | --- | --- | --- | --- | --- | --- | --- | --- | --- | --- | --- | --- | --- | --- | --- | --- | --- | --- | --- | --- | --- | --- | --- | --- | --- | --- | --- | --- | --- | --- | --- | --- | --- | --- | --- | --- | --- | --- | --- | --- | --- | --- | --- | --- | --- | --- | --- | --- | --- | --- | --- | --- | --- | --- | --- | --- | --- | --- | --- | --- | --- | --- | --- | --- | --- | --- | --- | --- | --- | --- | --- | --- | --- | --- | --- | --- | --- | --- | --- | --- | --- | --- | --- | --- | --- | --- | --- | --- | --- | --- | --- | --- | --- | --- | --- | --- | --- | --- | --- | --- | --- | --- | --- | --- | --- | --- | --- | --- | --- | --- | --- | --- | --- | --- | --- | --- | --- | --- | --- | --- | --- | --- | --- | --- | --- | --- | --- | --- | --- | --- | --- | --- | --- | --- | --- | --- | --- | --- | --- | --- | --- | --- | --- | --- | --- | --- | --- | --- | --- | --- | --- | --- | --- |

| *Supplementary Table 2: A Comparison of model performance for 6-month survival (≤182 days) in discovery and validation cohorts. B Comparison of 182-day OS in the discovery cohort for each of the scoring systems. Scottish MUO Score and CRP:Alb are continuous scales, and are grouped by decile. PLaCT score and mGPS utilise three prognostic groups. AUC Area Under the Curve, SE Standard Error, NPV Negative Predictive Value, PPV Positive Predictive Value*  *A*   \| **Prognostic Scoring System** \| AUC (SE) \| P-value pairwise comparison Scottish MUO Score \| Accuracy  (NPV) \| Precision  (PPV) \| Recall  (Sensitivity) \| Specificity \| \| --- \| --- \| --- \| --- \| --- \| --- \| --- \| \| *Discovery cohort* \| \| \| \| \| \| \| \| Scottish MUO Score \| 0.75  (0.02) \| - \| 0.58 \| 0.85 \| 0.13 \| 0.98 \| \| PlaCT \| 0.68  (0.02) \| 0.01 \| 0.79 \| 0.50 \| 0.93 \| 0.23 \| \| mGPS \| 0.55  (0.02) \| 5.77e-15 \| 0.80 \| 0.47 \| 0.97 \| 0.10 \| \| CRP:Alb \| 0.68 (0.02) \| 0.0002 \| 0.79 \| 0.51 \| 0.90 \| 0.29 \| \| *Validation cohort* \| \| \| \| \| \| \| \| Scottish MUO Score \| 0.69  (0.03) \| - \| 0.62 \| 0.62 \| 0.17 \| 0.93 \| \| PlaCT \| 0.69  (0.03) \| 0.94 \| 0.85 \| 0.47 \| 0.92 \| 0.29 \| \| mGPS \| 0.59  (0.03) \| 0.02 \| 0.77 \| 0.42 \| 0.95 \| 0.11 \| \| CRP:Alb \| 0.66  (0.03) \| 0.19 \| 0.78 \| 0.47 \| 0.87 \| 0.32 \|   B   \|  \| Scottish MUO Score \| \| CRP:Alb \| \|  \| PLaCT Score \| \| mGPS \| \| \| --- \| --- \| --- \| --- \| --- \| --- \| --- \| --- \| --- \| --- \| \| Decile \| N \| Died ≤182-days  N (%) \| N \| Died ≤182-days  N (%) \| Group \| N  (% of cohort) \| Died ≤182-days  N (%) \| N  (% of cohort) \| Died ≤182-days  N (%) \| \| 1 \| 60 \| 9 (15.0) \| 60 \| 10 (16.7) \| Good \| 96 (16.1) \| 20 (20.8) \| 40 (6.7) \| 8 (20.0) \| \| 2 \| 59 \| 13 (40.0) \| 59 \| 15 (25.4) \| \| 3 \| 60 \| 17 (22.0) \| 60 \| 21 (35.0) \| \| 4 \| 59 \| 18 (50.8) \| 59 \| 20 (33.9) \| Intermediate \| 268 (45.0) \| 98 (36.6) \| 150 (25.2) \| 67 (44.7) \| \| 5 \| 60 \| 23 (30.5) \| 60 \| 34 (56.7) \| \| 6 \| 59 \| 28 (47.5) \| 59 \| 28 (64.4) \| \| 7 \| 59 \| 32 (54.2) \| 59 \| 32 (47.5) \| Poor \| 231 (38.8) \| 151 (65.4) \| 405 (68.1) \| 194 (47.9) \| \| 8 \| 60 \| 40 (66.7) \| 60 \| 33 (55.0) \| \| 9 \| 59 \| 38 (64.4) \| 59 \| 35 (59.3) \| \| 10 \| 60 \| 51 (85.0) \| 60 \| 41 (68.3) \| |
| --- | --- | --- | --- | --- | --- | --- | --- | --- | --- | --- | --- | --- | --- | --- | --- | --- | --- | --- | --- | --- | --- | --- | --- | --- | --- | --- | --- | --- | --- | --- | --- | --- | --- | --- | --- | --- | --- | --- | --- | --- | --- | --- | --- | --- | --- | --- | --- | --- | --- | --- | --- | --- | --- | --- | --- | --- | --- | --- | --- | --- | --- | --- | --- | --- | --- | --- | --- | --- | --- | --- | --- | --- | --- | --- | --- | --- | --- | --- | --- | --- | --- | --- | --- | --- | --- | --- | --- | --- | --- | --- | --- | --- | --- | --- | --- | --- | --- | --- | --- | --- | --- | --- | --- | --- | --- | --- | --- | --- | --- | --- | --- | --- | --- | --- | --- | --- | --- | --- | --- | --- | --- | --- | --- | --- | --- | --- | --- | --- | --- | --- | --- | --- | --- | --- | --- | --- | --- | --- | --- | --- | --- | --- | --- | --- | --- | --- | --- | --- | --- | --- | --- | --- | --- | --- | --- | --- | --- | --- | --- | --- | --- | --- |

*Supplementary Table 3: A Comparison of model performance for 12-month survival (≤365 days) in discovery and validation cohorts. B Comparison of 365-day OS in the discovery cohort for each of the scoring systems. Scottish MUO Score and CRP:Alb are continuous scales, and are grouped by decile. PLaCT score and mGPS utilise three prognostic groups. AUC Area Under the Curve, SE Standard Error, NPV Negative Predictive Value, PPV Positive Predictive Value*

A

| **Prognostic Scoring System** | AUC (SE) | P-value pairwise comparison Scottish MUO Score | Accuracy  (NPV) | Precision  (PPV) | Recall  (Sensitivity) | Specificity |
| --- | --- | --- | --- | --- | --- | --- |
| *Discovery cohort* | | | | | | |
| Scottish MUO Score | 0.73  (0.02) | - | 0.41 | 0.93 | 0.10 | 0.99 |
| PlaCT | 0.67 (0.02) | 0.02 | 0.65 | 0.67 | 0.91 | 0.27 |
| mGPS | 0.53  (0.02) | 5.77e-13 | 0.63 | 0.63 | 0.96 | 0.11 |
| CRP:Alb | 0.67 (0.02) | 0.001 | 0.62 | 0.68 | 0.87 | 0.33 |
| *Validation cohort* | | | | | | |
| Scottish MUO Score | 0.68  (0.04) | - | 0.39 | 0.79 | 0.14 | 0.94 |
| PlaCT | 0.65 (0.03) | 0.52 | 0.56 | 0.68 | 0.86 | 0.31 |
| mGPS | 0.54  (0.03) | 0.001 | 0.45 | 0.64 | 0.93 | 0.11 |
| CRP:Alb | 0.64 (0.04) | 0.11 | 0.59 | 0.70 | 0.84 | 0.39 |

B

|  | Scottish MUO Score | | CRP:Alb | |  | PLaCT Score | | mGPS | |  |
| --- | --- | --- | --- | --- | --- | --- | --- | --- | --- | --- |
| Decile | N | Died ≤365-days  N (%) | N | Died ≤365-days  N (%) | Group | N  (% of cohort) | Died ≤365-days  N (%) | N  (% of cohort) | Died ≤365-days  N (%) |  |
| 1 | 60 | 17 (28.3) | 60 | 19 (31.7) | Good | 96 (16.1) | 34 (35.4) | 40 (6.7) | 15 (37.5) |  |
| 2 | 59 | 23 (40.0) | 59 | 25 (42.4) |  |  |  |  |  |  |
| 3 | 60 | 25 (41.7) | 60 | 30 (50.0) |  |  |  |  |  |  |
| 4 | 59 | 29 (49.2) | 59 | 33 (55.9) | Intermediate | 268 (45.0) | 152 (56.7) | 150 (25.2) | 95 (63.3) |  |
| 5 | 60 | 41 (68.3) | 60 | 45 (75.0) |  |  |  |  |  |  |
| 6 | 59 | 43 (72.9) | 59 | 38 (64.4) |  |  |  |  |  |  |
| 7 | 59 | 43 (72.9) | 59 | 44 (74.6) | Poor | 231 (38.8) | 181 (78.4) | 405 (68.1) | 257 (63.5) |  |
| 8 | 60 | 48 (80.0) | 60 | 42 (70.0) |  |  |  |  |  |  |
| 9 | 59 | 43 (72.9) | 59 | 45 (76.3) |  |  |  |  |  |  |
| 10 | 60 | 55 (91.7) | 60 | 46 (76.7) |  |  |  |  |  |  |
| *Supplementary Table 4: A Comparison of model performance for 30-day survival in discovery and validation cohorts. B Comparison of 30-day OS in the discovery cohort for each of the scoring systems. Scottish MUO Score and CRP:Alb are continuous scales, and are grouped by decile. PLaCT score and mGPS utilise three prognostic groups. AUC Area Under the Curve, SE Standard Error, NPV Negative Predictive Value, PPV Positive Predictive Value*  A   \| **Prognostic Scoring System** \| AUC (SE) \| P-value pairwise comparison Scottish MUO Score \| Accuracy  (NPV) \| Precision  (PPV) \| Recall  (Sensitivity) \| Specificity \| \| --- \| --- \| --- \| --- \| --- \| --- \| --- \| \| *Discovery cohort* \| \| \| \| \| \| \| \| Scottish MUO Score \| 0.74  (0.03) \| - \| 0.93 \| 0.37 \| 0.27 \| 0.95 \| \| PlaCT \| 0.73  (0.03) \| 0.93 \| 0.98 \| 0.11 \| 0.96 \| 0.17 \| \| mGPS \| 0.53  (0.03) \| 1.55e-5 \| 1.00 \| 0.10 \| 1.00 \| 0.07 \| \| CRP:Alb \| 0.71 (0.03) \| 0.30 \| 0.98 \| 0.11 \| 0.96 \| 0.22 \| \| *Validation cohort* \| \| \| \| \| \| \| \| Scottish MUO Score \| 0.73  (0.05) \| - \| 0.95 \| 0.14 \| 0.25 \| 0.90 \| \| PlaCT \| 0.68  (0.06) \| 0.50 \| 0.98 \| 0.07 \| 0.94 \| 0.21 \| \| mGPS \| 0.55  (0.06) \| 0.004 \| 0.95 \| 0.06 \| 0.94 \| 0.09 \| \| CRP:Alb \| 0.64  (0.07) \| 0.03 \| 0.98 \| 0.08 \| 0.94 \| 0.26 \|   B   \|  \| Scottish MUO Score \| \| CRP:Alb \| \|  \| PLaCT Score \| \| mGPS \| \| \| --- \| --- \| --- \| --- \| --- \| --- \| --- \| --- \| --- \| --- \| \| Decile \| N \| Died ≤30-days  N (%) \| N \| Died ≤30-days  N (%) \| Group \| N  (% of cohort) \| Died ≤30-days  N (%) \| N  (% of cohort) \| Died ≤30-days  N (%) \| \| 1 \| 60 \| 0 (0.0) \| 60 \| 0 (0.0) \| Good \| 96 (16.1) \| 2 (2.1) \| 40 (6.7) \| 0 (0.0) \| \| 2 \| 59 \| 2 (3.4) \| 59 \| 2 (3.4) \| \| 3 \| 60 \| 3 (5.0) \| 60 \| 2 (3.3) \| \| 4 \| 59 \| 2 (3.4) \| 59 \| 3 (5.1) \| Intermediate \| 268 (45.0) \| 9 (3.4) \| 150 (25.2) \| 16 (10.7) \| \| 5 \| 60 \| 5 (8.3) \| 60 \| 6 (10.0) \| \| 6 \| 59 \| 4 (6.8) \| 59 \| 7 (11.9) \| \| 7 \| 59 \| 7 (11.9) \| 59 \| 6 (10.2) \| Poor \| 231 (38.8) \| 45 (19.5) \| 405 (68.1) \| 40 (9.9) \| \| 8 \| 60 \| 8 (13.3) \| 60 \| 8 (13.3) \| \| 9 \| 59 \| 7 (11.9) \| 59 \| 10 (16.9) \| \| 10 \| 60 \| 18 (30.0) \| 60 \| 12 (20.0) \| | | | | | | | | | | |

**References**

1. Therneau TM. A Package for Survival Analysis in S, Version 2.38 2015 [Available from: <https://CRAN.R-project.org/package=survival>.

2. Kassambara AK, M. survminer: Drawing Survival Curves using 'ggplot2'. R package version 0.4.4 2019 [Available from: <https://CRAN.R-project.org/package=survminer>.

3. Harrison E, Drake T, Ots R. finalfit: Quickly Create Elegant Regression Results Tables and Plots when Modelling. [R package]. 2020 [version 1.0.2:[Available from: <https://CRAN.R-project.org/package=finalfit>.

4. Kowarik AT, M. . Imputation with the R Package VIM. J Statistical Software. 2016;74(7):1-16.

5. Venables WN, Ripley B.D. Modern Applied Statistics with S. Fourth Edition. Springer, New York. 2002 [

6. Kuhn M. Classification and Regression Training. R package version 6.0-92 2022 [Available from: <https://CRAN.R-project.org/package=caret>.

7. Izumi K, Mizokami A, Maeda Y, Koh E, Namiki M. Current outcome of patients with ureteral stents for the management of malignant ureteral obstruction. J Urol. 2011;185(2):556-61.

8. Izumi K, Shima T, Shigehara K, Sawada K, Naito R, Kato Y, et al. A novel risk classification score for malignant ureteral obstruction: a multicenter prospective validation study. Sci Rep. 2021;11(1):4455.

9. McMillan DC. The systemic inflammation-based Glasgow Prognostic Score: a decade of experience in patients with cancer. Cancer Treat Rev. 2013;39(5):534-40.

10. McMillan DC, Crozier JE, Canna K, Angerson WJ, McArdle CS. Evaluation of an inflammation-based prognostic score (GPS) in patients undergoing resection for colon and rectal cancer. Int J Colorectal Dis. 2007;22(8):881-6.

11. Sachs MC. plotROC: A Tool for Plotting ROC Curves. J Stat Softw. 2017;79.

12. Robin X, Turck N, Hainard A, Tiberti N, Lisacek F, Sanchez JC, et al. pROC: an open-source package for R and S+ to analyze and compare ROC curves. BMC Bioinformatics. 2011;12:77.

13. Cordeiro MD, Coelho RF, Chade DC, Pessoa RR, Chaib MS, Colombo-Junior JR, et al. A prognostic model for survival after palliative urinary diversion for malignant ureteric obstruction: a prospective study of 208 patients. BJU Int. 2016;117(2):266-71.
